# Mottling as a prognosis marker in cardiogenic shock

**DOI:** 10.1186/s13613-023-01175-0

**Published:** 2023-09-06

**Authors:** Hamid Merdji, Vincent Bataille, Anais Curtiaud, Laurent Bonello, François Roubille, Bruno Levy, Pascal Lim, Francis Schneider, Hadi Khachab, Jean-Claude Dib, Marie-France Seronde, Guillaume Schurtz, Brahim Harbaoui, Gerald Vanzetto, Severine Marchand, Caroline Eva Gebhard, Patrick Henry, Nicolas Combaret, Benjamin Marchandot, Benoit Lattuca, Caroline Biendel, Guillaume Leurent, Edouard Gerbaud, Etienne Puymirat, Eric Bonnefoy, Ferhat Meziani, Clément Delmas

**Affiliations:** 1 Faculté de Médecine, Strasbourg University Hospital, Nouvel Hôpital Civil, Medical Intensive Care Unit, Université de Strasbourg (UNISTRA), Strasbourg, France; 2 Department of Cardiology, Toulouse Rangueil University Hospital, UMR 1295 INSERM, Toulouse, France; 3 Aix-Marseille Université, 13385 Marseille, France; 4 Intensive Care Unit, Department of Cardiology, Assistance Publique-Hôpitaux de Marseille, Hôpital Nord, 13385 Marseille, France; 5 Mediterranean Association for Research and Studies in Cardiology (MARS Cardio), Marseille, France; 6 PhyMedExp, Université de Montpellier, INSERM, CNRS, Cardiology Department, INI-CRT, CHU de Montpellier, Montpellier, France; 7 CHRU Nancy, Réanimation Médicale Brabois, Vandoeuvre-les Nancy, France; 8 Univ Paris Est Créteil, INSERM, IMRB, 94010 Créteil, France; 9 AP-HP, Hôpital Universitaire Henri-Mondor, Service de Cardiologie, 94010 Créteil, France; 10 Médecine Intensive-Réanimation, Hôpital de Hautepierre, Hôpitaux Universitaires de Strasbourg, Strasbourg, France; 11 Intensive Cardiac Care Unit, Department of Cardiology, CH d’Aix en Provence, Aix-en-Provence, France; 12 Avenue des Tamaris, 13616 Aix-en-Provence cedex 1, France; 13 Clinique Ambroise Paré, Neuilly-sur-Seine, France; 14 Service de Cardiologie CHU Besançon, Besançon, France; 15 Urgences et Soins Intensifs de Cardiologie, CHU Lille, University of Lille, Inserm U1167, 59000 Lille, France; 16 Cardiology Department, Hôpital Croix-Rousse and Hôpital Lyon Sud, Hospices Civils de Lyon, Lyon, France; 17 University of Lyon, CREATIS UMR5220, INSERM U1044, INSA-15, Lyon, France; 18 Department of Cardiology, Hôpital de Grenoble, 38700 La Tronche, France; 19 Service de Cardiologie, Clinique Mutualiste, Grenoble, France; 20 Intensive Care Unit, Department of Acute Medicine, University Hospital Basel, Petersgraben 4, 4031 Basel, Switzerland; 21 Department of Cardiology, AP-HP, Lariboisière University Hospital, Paris, France; 22 Department of Cardiology, CHU Clermont-Ferrand, CNRS, Université Clermont Auvergne, Clermont-Ferrand, France; 23 Université de Strasbourg, Pôle d’Activité Médico-Chirurgicale Cardio-Vasculaire, Nouvel Hôpital Civil, Centre Hospitalier Universitaire, 67091 Strasbourg, France; 24 Department of Cardiology, Nîmes University Hospital, Montpellier University, Nîmes, France; 25 Intensive Cardiac Care Unit, Rangueil University Hospital, 1 Avenue Jean Poulhes, 31059 Toulouse Cedex, France; 26 Institute of Metabolic and Cardiovascular Diseases (I2MC), UMR-1048, National Institute of Health and Medical Research (INSERM), Toulouse, France; 27 Department of Cardiology, CHU Rennes, Inserm, LTSI—UMR 1099, Univ Rennes 1, 35000 Rennes, France; 28 Intensive Cardiac Care Unit and Interventional Cardiology, Hôpital Cardiologique du Haut Lévêque, 5 Avenue de Magellan, 33604 Pessac, France; 29 Bordeaux Cardio-Thoracic Research Centre, U1045, Bordeaux University, Hôpital Xavier Arnozan, Avenue du Haut Lévêque, 33600 Pessac, France; 30 Department of Cardiology, Assistance Publique-Hôpitaux de Paris (AP-HP), Hôpital Européen Georges Pompidou, 75015 Paris, France; 31 Université de Paris, 75006 Paris, France; 32 Intensive Cardiac Care Unit, Lyon Bron University Hospital, Lyon, France; 33 Recherche et Enseignement en Insuffisance Cardiaque Avancée Assistance et Transplantation (REICATRA), Institut Saint Jacques, CHU Toulouse, Toulouse, France

**Keywords:** Cardiogenic shock, Acute heart failure, Perfusion, Microcirculation

## Abstract

**Aims:**

Impact of skin mottling has been poorly studied in patients admitted for cardiogenic shock. This study aimed to address this issue and identify determinants of 30-day and 1-year mortality in a large cardiogenic shock cohort of all etiologies.

**Methods and results:**

FRENSHOCK is a prospective multicenter observational registry conducted in French critical care units between April and October, 2016. Among the 772 enrolled patients (mean age 65.7 ± 14.9 years; 71.5% male), 660 had skin mottling assessed at admission (85.5%) with almost 39% of patients in cardiogenic shock presenting mottling. The need for invasive respiratory support was significantly higher in patients with mottling (50.2% vs. 30.1%, *p* < 0.001) and likewise for the need for renal replacement therapy (19.9% vs. 12.4%, *p* = 0.09). However, the need for mechanical circulatory support was similar in both groups. Patients with mottling at admission presented a higher length of stay (19 vs. 16 days, *p* = 0.033), a higher 30-day mortality rate (31% vs. 23.3%, *p* = 0.031), and also showed significantly higher mortality at 1-year (54% vs. 42%, *p* = 0.003). The subgroup of patients in whom mottling appeared during the first 24 h after admission had the worst prognosis at 30 days.

**Conclusion:**

Skin mottling at admission in patients with cardiogenic shock was statistically associated with prolonged length of stay and poor outcomes. As a perfusion-targeted resuscitation parameter, mottling is a simple, clinical-based approach and may thus help to improve and guide immediate goal-directed therapy to improve cardiogenic shock patients’ outcomes.

**Graphical Abstract:**

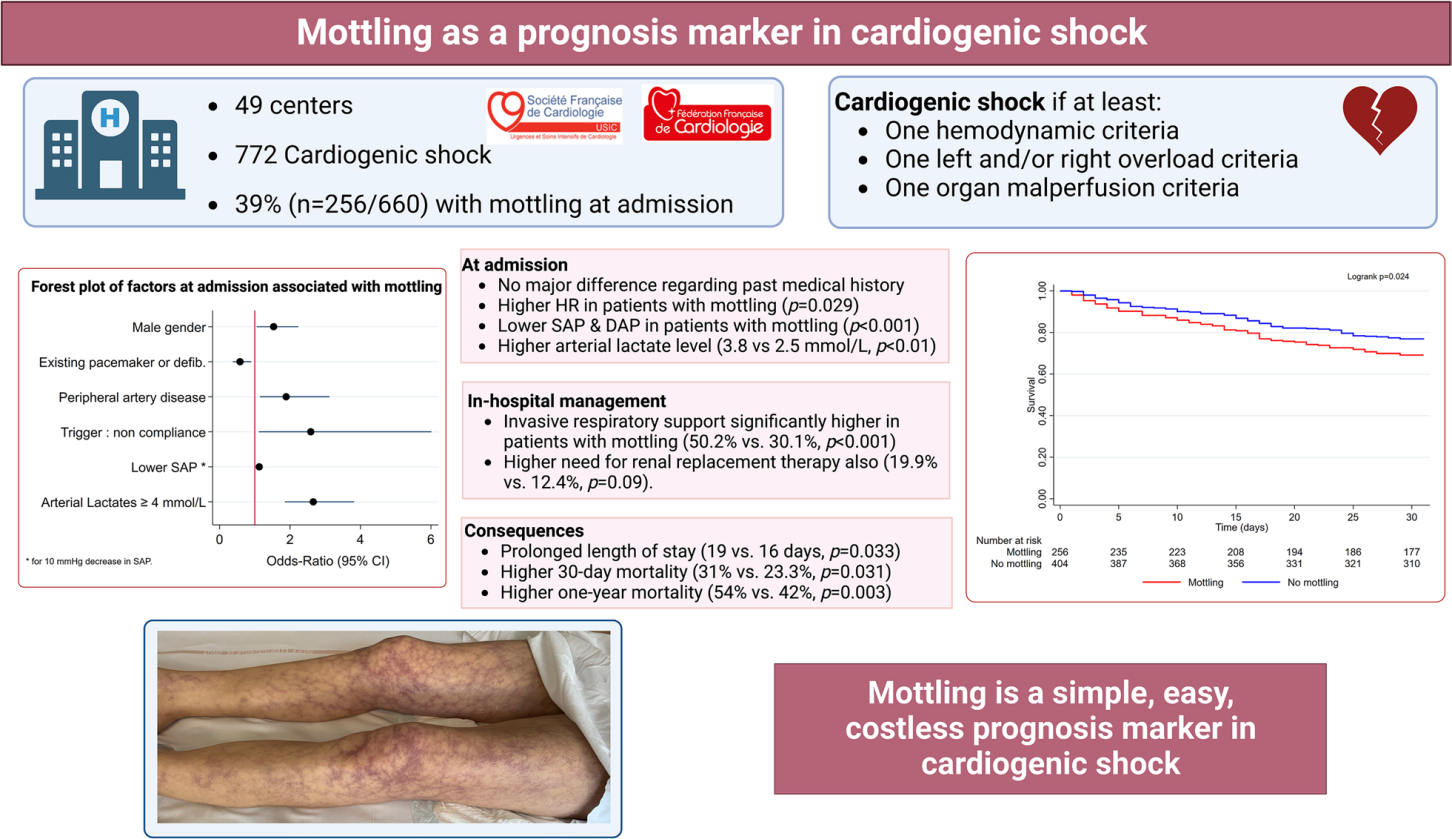

**Supplementary Information:**

The online version contains supplementary material available at 10.1186/s13613-023-01175-0.

## Introduction

To date, even if there is no precise uniform definition of cardiogenic shock (CS), it is generally considered as a primary cardiac dysfunction with low cardiac output leading to critical end-organ hypoperfusion [1, 2] with a high mortality rate (40%) [3]. This inadequate end-organ perfusion associated with microcirculatory dysfunction and multiple organ failure is mentioned in all current definitions of CS as “*signs of poor peripheral tissue perfusion*” such as cold extremities, mottling, elevated capillary refill time (CRT), altered mental status, oliguria or elevated arterial lactate levels [4]. However, even if the classification of acute heart failure (AHF) and CS patients using perfusion/congestion profiles for treatment approaches related to each category are suggested by the latest European Society of Cardiology guidelines [5] and considered as class I recommendation by the American College of Cardiology/American Heart Association Joint Committee latest guidelines [6], the level of evidence is low (C) due to limited data [6]. Indeed, only recently have studies attempted to better characterize the hypoperfusion and microcirculatory dysfunction in CS [7–9].

Numerous investigations have provided evidence that CS affects not only the macrocirculation, as evidenced by alterations in blood pressure (BP), left ventricular ejection fraction (LVEF), and cardiac index (CI) [10–13], but also has significant perfusion abnormalities of the systemic microcirculation [14, 15]. This intricate network of microvessels, arterioles, capillaries, and venules, which constitutes the terminal vascular network of the systemic circulation, plays a crucial role in the delivery of oxygen and nutrients to organs, as well as the removal of carbon dioxide [16].

The visualization of microcirculation can be achieved using handheld microscopes, including Sidestream Dark-Field (SDF), Incident Dark-Field (IDF) imaging techniques, and tissue laser Doppler imaging [17]. However, these devices have several limitations, such as the need for trained operators, limited availability due to the expensive cost of these devices, difficulty evaluating the sublingual area in non-intubated patients, and limited representation of microcirculatory impairment in other tissues [18]. Interestingly, traditional markers of peripheral tissue perfusion, including skin mottling and CRT, are strongly associated with altered microcirculatory blood flow during septic shock [19–21]. Mottling refers to patchy discoloration of the skin, usually starting around the knees, while CRT measures the time required to recolor the tip of a finger [22].

Even if mottling is easy to assess, costless, and widely described and taught in medical school as a sign of shock or hypoperfusion for centuries, they have been poorly studied in CS to date [7]. Indeed, only one small study has rigorously investigated them in CS [9].

The main objective of this study based on the largest European prospective cohort of CS to date was to assess characteristics and outcomes of CS according to the presence of mottling at admission. The secondary objectives were to assess morbidity and mortality parameters, and also the time course of mottling contribution to CS outcomes among the subgroup of patients who survived the first 24 h.

## Methods

### Patient population

FRENSHOCK is a prospective multicenter observational registry conducted in metropolitan France during 6 months between April and October 2016 in intensive care units (ICU) and intensive cardiac care units (ICCU) (NCT02703038). The methods used for this registry have been previously described [23]. Briefly, the primary objective was to evaluate the characteristics, management, and outcomes of CS patients, with a new modified definition of CS as seen in routine clinical practice, on a nationwide scale.

All adult patients (≥ 18 years old) with CS were prospectively included in this registry if they met at least one criterion of each of the following three components: (i) hemodynamic criteria, defined as low systolic arterial pressure (SAP) < 90 mmHg and/or the need for maintenance with vasopressors/inotropes and/or a low CI < 2.2 L/min/m^2^; (ii) left and/or right heart overload, defined by clinical signs, radiology, blood tests, echocardiography, or invasive hemodynamics’ signs; and (iii) signs of organ malperfusion, which could be clinical and/or biological. Patients admitted after cardiopulmonary resuscitation were included if they fulfilled previously defined CS criteria. Patients could be included regardless of CS etiology, and whether CS was primary or secondary. Exclusion criteria were refusal or the inability to consent. A diagnosis of CS was refuted in favor of alternative diagnoses, such as septic shock, refractory cardiac arrest, and post-cardiotomy CS [23].

All institutions were invited to participate in the study, including university teaching hospitals, general and regional hospitals, as well as public and private hospitals that manage CS patients (ICCUs, surgical ICUs, medical ICUs, and general ICUs).

The study was conducted in accordance with the guidelines for good clinical practice and French law. Written consent was obtained for all the patients. The data recorded and their handling and storage were reviewed and approved by the CCTIRS (French Health Research Data Processing Advisory Committee) (n° 15.897) and the CNIL (French Data Protection Agency) (n° DR-2016-109).

### Data collection

Data on baseline characteristics, including demographics (age, gender, body mass index, social status), risk factors (hypertension, diabetes, current smoking, hypercholesterolemia, family history of coronary artery disease), and medical history [cardiomyopathy, myocardial infarction, stroke, peripheral artery disease, chronic kidney disease, active cancer, chronic obstructive lung disease], were collected as previously mentioned. Clinical, biological, and echocardiographic data were collected at admission and 24 h after admission. Skin mottling, define as patchy skin discoloration starting around the knees was assessed at admission and 24 h after admission by a senior physician as requested in the case report form. Therefore, patients with mottling (of any extent) were considered in the group called “Mottling” and those without mottling in the "No mottling" group. Up to three CS triggers were determined for each patient by the local investigator, that is, ischemic (Type 1 or Type 2 acute myocardial infarction according to European guidelines); ventricular and supraventricular arrhythmia; conduction disorder; infectious disease; non-compliance (poor compliance with medical treatment or hygiene and diet rules, for example, stopping or skipping an angiotensin-converting enzyme inhibitor or beta-blocker treatment, deviation from a low sodium diet, etc.); or iatrogenesis. Investigators could also note other existing factors or etiologies. Such triggering factors were indicated as ‘other’. Information regarding the use of cardiac procedures, that is, coronary angiography and/or percutaneous coronary intervention (PCI); right heart catheterization; the need for medications (inotropes, vasopressors, diuretics, and fibrinolysis) and organ replacement therapies such as mechanical ventilation (invasive or non-invasive); temporary mechanical circulatory support [intra-aortic balloon pump (IABP); venoarterial-extracorporeal membrane oxygenation (VA-ECMO) or Impella^®^ (Abiomed, Danvers, MA, USA)]; and renal replacement therapy (RRT) were collected. In-hospital complications were noted, such as stroke, bleeding and transfusions, hemolysis, thrombocytopenia, nosocomial infections, vascular complications, and death. Information on mortality was obtained directly by the local investigators (cause and date) through a 30-day and 1-year follow-up.

### Statistical analysis

Continuous variables were reported as means (standard deviation, SD) or medians and interquartile ranges when appropriate. Discrete variables were described in numbers and percentages. The two groups (presence or absence of mottling at admission) were compared using student’s *t* tests or Mann and Whitney non-parametric tests for continuous variables and using *χ*^2^ or Fisher’s exact tests for categorical variables. Factors independently associated with mottling were studied using multiple logistic regression. Survival analyses were conducted using the Kaplan–Meier method and factors associated with 30 days and 1-year mortality were identified using a Cox Proportional Hazards Model, with a stepwise backward method for covariates elimination. Odds ratios (ORs) and hazard ratios (HRs) were presented with their 95% confidence intervals (CIs). Statistical analyses were performed using Stata (Stata Statistical Software SE/17.0. StataCorp LLC. College Station. TX. USA.). For all analyses, two-sided *p* values < 0.05 were considered significant.

## Results

### Study population

A total of 772 CS patients were included in 49 centers, in whom mottling was assessed in 660 patients at inclusion (85.5%). Among these CS patients, clinical characteristics between patients with and without mottling are presented in Table 1. The mean age (66 ± 14 years) was similar in the two groups, but men were significantly predominant in mottling patients (76% vs. 68%, *p* = 0.02). In patients with mottling, a medical history of cardiac disease was reported in 53.9% (30.9% coronary artery disease), previous PCI in 21.9%, previous ischemic stroke in 9.8%, and peripheral artery disease in 15.2% with no significant difference between groups. There was also no difference in terms of cardiovascular risk factors, or medical history except for a higher rate of already implanted pacemakers or defibrillators among patients with mottling (*p* = 0.042 and *p* = 0.041, respectively). Peripheral artery disease was numerically more frequent among patients with mottling (15.2% vs. 10.2%).

**Table 1:** Baseline characteristics of cardiogenic shock patients included

	Overall (*n* = 660)		No mottling (*n* = 404)		Mottling (*n* = 256)		*p*
Male gender	469	71.1	275	68.1	194	75.8	0.020
Age (years), mean ± SD	66.0	± 14.0	65.9	± 15.7	66.0	± 14.0	0.963
BMI (kg/m^2^), mean ± SD	25.8	± 5.6	25.8	± 5.9	25.7	± 4.9	0.977
*n*	*644*		*394*		*250*		
Risk factors, *n* (%)							
Current smoker	182/634	28.7	107/387	27.7	75/247	30.4	0.461
Diabetes mellitus	183/659	27.8	115/403	28.5	68/256	0.6	0.581
Arterial hypertension	320	48.5	190	47.0	130	50.8	0.347
Dyslipidaemia	236	35.8	137	33.9	99	38.7	0.214
Medical history, *n* (%)							
History of cardiac disease	368	55.8	230	56.9	138	53.9	0.446
Ischaemic	197	29.9	118	29.2	79	30.9	0.651
Hypertrophic	10	1.5	7	1.7	3	1.2	0.748
Idiopathic	65	9.9	47	11.6	18	7.0	0.053
Toxic	26	3.9	12	3.0	14	5.5	0.108
Multisite pacing	51	7.7	38	9.4	13	5.1	0.042
Defibrillator	104	15.8	73	18.1	31	12.1	0.041
CABG	54	8.2	36	8.9	18	7.0	0.391
PCI	145	22.0	89	22.0	56	21.9	0.963
Peripheral artery disease	80	12.1	41	10.2	39	15.2	0.051
Ischaemic stroke	56	8.5	31	7.7	25	9.8	0.34.7
Chronic renal failure	139	21.1	88	21.8	51	19.9	0.568
COPD	45	6.8	28	6.9	17	6.6	0.885
Active neoplasy	43	6.5	22	5.5	21	8.2	0.162
Previous medications, *n* (%)							
Aspirin	250/659	37.9	157/404	38.9	93/255	36.5	0.538
P2Y12 inhibitor	119/659	18.1	77/404	19.1	42/255	16.5	0.400
Statins	243/659	36.9	154/404	38.1	89/255	34.9	0.404
Beta-blockers	273/659	41.4	177/404	43.8	96/255	41.4	0.118
Vitamin K antagonist	144/659	21.9	93/404	23.0	51/255	20.0	0.361
Direct oral anticoagulant	50/659	7.6	29/404	7.2	21/255	8.2	0.618
ACE inhibitors or ARB	248/659	37.6	154/404	38.1	94/255	36.9	0.746
Sacubitril/valsartan	15/659	2.4	9/404	2.3	6/255	2.5	1.000
Furosemide	325/659	49.3	210/404	52.0	115/255	45.1	0.085
Aldosterone antagonist	94/659	14.3	67/404	16.6	27/255	10.6	0.032
Amiodarone	119/643	18.5	66/391	16.9	53/252	21.0	0.186
Proton pump inhibitor	236/650	36.3	152/397	38.3	84/253	33.2	0.189
Triggers							
Ischaemic	255	38.6	151	37.4	104	40.6	0.404
Mechanical	17	2.6	8	2.0	9	3.5	0.225
Ventricular arrhythmia	83	12.6	45	11.1	38	14.8	0.162
Atrial arrhythmia	95	14.4	56	13.9	39	15.2	0.624
Conductive disorders	17	2.6	10	2.5	7	2.7	0.838
Infectious	80	12.1	45	11.1	35	13.7	0.331
Non-compliance	26	3.9	10	2.5	16	6.3	0.015
Iatrogenic	49	7.4	29	7.2	20	7.8	0.762
Other	88	13.3	57	14.1	31	12.1	0.462
None/undefined	92	13.9	67	16.6	25	9.8	0.783

Values in italics indicate the number of patients included in the statistical analysis

ACE, angiotensin-converting enzyme; ARB, angiotensin-receptor blocker; BMI, body mass index; CABG, coronary artery bypass graft; COPD, chronic obstructive pulmonary disease; PCI, percutaneous coronary intervention; SD, standard deviation

There was no significant difference in previous cardiac treatments except more aldosterone antagonists being prescribed in non-mottling patients (*p* = 0.032). Besides cardiovascular diseases, there was also no difference in medical history of chronic obstructive pulmonary disease or chronic kidney disease.

At admission, in patients with mottling, the mean heart rate was 99 (± 35.2) bpm (47.3% with sinus rhythm), SAP was 97 (± 27) mmHg, and DAP was 60 (± 18) mmHg. Heart rate was statistically higher in CS patients with mottling while blood pressure parameters were statistically lower compared to CS patients without mottling (*p* = 0.029, *p* < 0.01, and *p* < 0.01, respectively) (Table 2). No significant difference was found between patients with and patients without mottling regarding clinical signs of left (75% vs. 68%, respectively, *p* = 0.18) and right (53% vs. 47%, *p* = 0.13) heart failure.

**Table 2:** Clinical, echographic, and biological characteristics of cardiogenic shock patients included

	Overall (*n* = 660)		No mottling (*n* = 404)		Mottling (*n* = 256)		*p*
Admission unit, *n* (%)							0.007
CICU	356	69.1	235	73.4	121	62.1	
Reanimation	159	30.9	85	26.6	74	37.9	
Clinical presentation at admission							
Heart rate (bpm), mean ± SD	95.8	± 30.0	93.8	± 26.0	99	± 35.2	0.029
*n*	*659*		*403*		*256*		
SAP (mmHg), mean ± SD	101	± 25	103	± 23	97	± 27	< 0.001
*n*	*660*		*404*		*256*		
DAP (mmHg), mean ± SD	63	± 18	65	± 17	60	± 18	< 0.001
*n*	*659*		*403*		*256*		
Sinus rhythm, *n* (%)	336/659	51.0	215/403	53.4	121/256	47.3	0.128
Cardiac arrest, *n* (%)	73	11.1	38	9.4	35	13.7	0.089
Blood tests at admission							
Sodium (mmol/L), mean ± SD	135	± 6	135	± 6	135	± 6	0.608
*n*	*652*		*399*		*253*		
eGFR (mL/min/1.73 m^2^), mean ± SD	49.5	± 26.2	50.8	± 27.6	47.4	± 23.8	0.112
*n*	*644*		*396*		*248*		
Bilirubin (mg/L), median (IQR)	16 (9–28)		16 (9–27)		17 (10–32)		0.159
*n*	*461*		*281*		*180*		
Hamoglobin (g/dL), mean ± SD	12.5	± 2.5	12.5	± 2.3	12.5	± 2.5	0.808
*n*	*649*		*398*		*251*		
Arterial blood lactates (mmol/L), median (IQR)	3.0 (2.0–5.0)		2.5 (2.0–4.0)		3.8 (2.0–6.0)		< 0.001
*n*	*595*		*357*		*238*		
ASAT (IU/L), median (IQR)	91 (38–304)		83 (37–270)		118 (42–388)		0.077
*n*	*458*		*288*		*170*		
ALAT (IU/L), median (IQR)	59 (26–184)		57 (25–179)		69 (31–236)		0.074
*n*	*468*		*292*		*176*		
Nt proBNP (pg/mL), median (IQR)	8938 (3894–24,363)		8388 (3466–20,333)		9277 (4411–30,000)		0.399
*n*	*185*		*111*		*74*		
BNP (pg/mL), median (IQR)	1211 (484–2852)		1437 (509–2997)		1099 (484–2262)		0.236
*n*	*234*		*153*		*81*		
CRP (mg/L), median (IQR)	28 (10–64)		26 (9–56)		32 (10–95)		0.111
*n*	*352*		*225*		*127*		
Baseline echography							
LVEF (%), mean ± SD	26.2	± 13.2	26.8	± 12.6	25.3	± 14.1	0.160
*n*	*653*		*400*		*253*		
TAPSE (mm), mean ± SD	13.5	± 5.1	13.9	± 5.1	12.9	± 5.2	0.132
*n*	*235*		*148*		*87*		
PSVtdi (cm/s), median (IQR)	8 (6–11)		8 (6–11)		9 (6–11)		0.443
*n*	*191*		*121*		*70*		
Severe mitral regurgitation, *n* (%)	94/631	14.9	64/388	16.5	30/243	12.4	0.154
Severe aortic stenosis, *n* (%)	32/650	4.9	14/399	3.5	18/251	7.2	0.036
Severe aortic regurgitation, *n* (%)	9/647	1.4	4/398	1.0	5/249	2.0	0.316

Values in italics indicate the number of patients included in the statistical analysis

ALAT, alanine aminotransferase; ASAT, aspartate aminotransferase; CICU, cardiologic intensive care unit; CRP, C-reactive protein; DAP, diastolic arterial pressure; IQR, interquartile range; LVEF, left ventricular ejection fraction; PSVtdi, peak systolic velocity tissue Doppler imaging; SAP, systolic arterial pressure; SD, standard deviation; TAPSE, tricuspid annular plane systolic excursion

The main triggers of CS (not mutually exclusive) in patients with mottling were ischemic (40.6%), atrial arrhythmia (15.2%), and ventricular arrhythmia (14.8%) (Table 1). Non-compliance to cardiovascular medications was significantly more frequent in patients with mottling (6.3% vs. 2.5%, *p* = 0.015) compared to patients without mottling. Most patients in both groups had multiple organ failures as evidenced by kidney dysfunction, hepatic cytolysis and cholestasis, and lactate elevation (Table 2). Notably, lactate level at admission was significantly increased in patients with mottling compared to patients without (3.8 vs. 2.5 mmol/L, *p* < 0.01).

At baseline echocardiography, patients with mottling showed a mean left ventricular ejection fraction (LVEF) of 25.3% (± 14.1) which was not different from patients without mottling. Severe aortic stenosis appeared to be almost twice more frequent in patients with mottling (7.2% vs. 3.5%, *p* = 0.036).

### Factors associated with mottling

A multivariate analysis identified four independent factors at admission associated with mottling: male gender (OR: 1.5; *p* = 0.025), peripheral artery disease (OR: 1.9; *p* = 0.013), non-compliance as a trigger (OR: 2.6; *p* = 0.027), and lactates > 4 mmol/L (OR: 2.7; *p* < 0.001) (Table 3).

**Table 3:** Baseline characteristics associated with mottling

	Odds-ratio	95% CI	*p*
Male gender	1.53	1.05–2.23	0.025
Existing pacemaker of defibrillator	0.58	0.37–0.90	0.015
Peripheral artery disease	1.89	1.14–3.12	0.013
Trigger: non-compliance	2.59	1.11–6.01	0.027
SAP (mmHg)	0.89	0.83–0.95	0.001
Lactates (mmol/L)			
< 4	1.00	Ref.	
4 +	2.66	1.85–3.82	< 0.001
Unknown	0.94	0.52–1.72	0.848

Hosmer and Lemeshow goodness of fit *p* = 0.573

CI, confidence interval; SAP, systolic arterial pressure

### In-hospital management

In-hospital management is reported in Table 4. Approximately 70% of the CS patients were directly referred to ICCU while 30% were directly referred to ICU*.*

**Table 4:** In-hospital management and prognosis of cardiogenic shock patients according to the presence of mottling at admission

	Overall (*n* = 660)		No mottling (*n* = 404)		Mottling (*n* = 256)		*p*
Medications used, *n* (%)							
Diuretics	532/657	81.0	332/402	82.6	200/255	78.4	0.186
Volume expander	281/656	42.8	153/401	38.2	128/255	50.2	0.002
Dobutamine	540/657	82.2	329/402	81.8	211/255	82.8	0.768
If yes, maximum dose (mg/kg/min)							0.007
5–10	340	62.6	225	68.0	115	54.2	
10–15	121	22.3	59	17.8	62	29.2	
> 15	44	8.1	26	7.9	18	8.5	
Unknown	38	7.0	21	6.3	17	8.0	
Norepinephrine	344/657	52.4	194/402	48.3	150/255	58.8	0.008
If yes, maximum dose (mg/h)							0.128
< 1	67	19.3	44	22.4	23	15.2	
1–5	177	51.0	99	50.5	78	51.7	
> 5	71	20.5	33	16.8	38	25.2	
Unknown	32	9.2	20	10.2	12	7.9	
Epinephrine	86/657	13.1	39/402	9.7	47/255	18.4	0.001
If yes, maximum dose (mg/h)							0.395
< 1	32	36.0	18	43.9	14	29.2	
1–5	34	38.2	12	29.3	22	45.8	
> 5	13	14.6	6	14.6	7	14.6	
Unknown	10	11.2	5	12.2	5	10.4	
Norepinephrine + dobutamine combination	299/657	45.5	165/402	41.0	134/255	52.6	0.004
Levosimendan	47/657	7.2	34/402	8.5	13/255	5.1	0.576
Dopamine	1/657	0.2	0/402	0.0	1/255	0.4	1.000
Isoprenaline	30/657	4.6	15/402	3.7	15/255	5.9	0.198
Antiarrhythmic	261/657	39.7	154/402	38.3	107/255	42.0	0.351
Transfusion	115/656	17.5	60/401	15.0	55/255	21.6	0.030
Fibrinolysis	11/656	1.7	5/401	1.3	6/255	2.4	0.353
Organ replacement therapies, *n* (%)							
Respiratory support							
Invasive	249/657	37.9	121/402	30.1	128/255	50.2	< 0.001
Non-invasive	183/657	27.9	103/402	25.6	80/255	31.4	0.109
Mechanical circulatory support	122/658	18.5	66/402	16.4	56/256	21.9	0.079
If yes							
IABP	43/121	35.5	23/66	34.9	20/55	36.4	0.862
Impella	22/121	18.2	11/66	16.7	11/55	20.0	0.636
ECLS	72/121	59.5	37/66	56.1	35/55	63.6	0.398
Renal replacement therapy	101/659	15.3	50/403	12.4	51/256	19.9	0.009
Invasive cardiology, *n* (%)							
CAG	346	52.4	219	54.2	127	49.6	0.249
If yes							
CAG result							0.205
Normal	61	17.6	39	17.8	22	17.3	
1—Mono	71	20.5	51	23.3	20	15.7	
2—Bi	80	23.1	49	22.4	31	24.4	
3—Tri	76	22.0	50	22.8	26	20.5	
Unknown	58	16.8	30	13.7	28	22.0	
Culprit lesion	225/281	80.1	143/178	80.3	82/103	79.6	0.883
Any PCI	192	29.1	121	30.0	71	27.7	0.541
Right heart catheterisation	107/657	16.3	70/402	17.4	37/255	14.5	0.326
Pace-maker implantation	28/625	4.5	16/388	4.1	12/237	5.1	0.582
Defibrillator implantation	31/625	5.0	18/388	4.6	13/237	5.5	0.636
Radiofrequency ablation	15/625	2.4	13/388	3.4	2/237	0.8	0.047
Discharge							
LVEF (%), mean ± SD	34.8	± 14.2	34.2	± 13.7	36	± 14.9	0.221
*n*	*375*		*240*		*135*		
LVEF variation*, mean ± SD	8.2	± 14.0	7.4	± 13.7	9.5	± 14.5	0.172
*n*	*372*		*238*		*134*		
Length of stay in ICU/ICCU (days), median (IQR)	12 (7–21)		11 (7–20)		13 (8–25)		0.061
*n*	*380*		*232*		*148*		
Length of stay in hospital (days), median (IQR)	16 (11–27)		16 (11–25)		19 (12–31)		0.033
*n*	*367*		*238*		*129*		
Discharge mode							0.074
Home	141	25.4	96	28.3	45	20.8	
Rehabilitation	38	6.8	23	6.8	15	6.9	
Transferred (other center/other department)	183	33.0	116	34.2	67	31.0	
Death	190	34.2	103	30.4	87	40.3	
Other	3	0.5	1	0.3	2	0.9	
Registration on transplant waiting list	33/552	6.0	21/333	6.3	12/219	5.5	0.689
Mortality							
30-day mortality	173	26.2	94	23.3	79	30.9	0.031
1-year mortality**	308	46.7	170	42.1	138	53.9	0.003

Values in italics indicate the number of patients included in the statistical analysis

CAG, coronary arteriography; ECLS, extracorporeal membrane oxygenation; IABP, intra-aortic balloon pump; ICCU, intensive cardiac care unit; ICU, intensive care unit; IQR, interquartile range; LVEF, left ventricular ejection fraction; PCI, percutaneous coronary intervention; SD, standard deviation

*At discharge compared with admission

**2.5% of patients lost to follow-up at 1-year

Medical management was relatively different between patients with mottling and patients without. Patients with mottling had significantly more volume expansion (*p* = 0.002), more maximum dose of dobutamine above 10 µg/kg/min (*p* = 0.007), more often norepinephrine (*p* = 0.008), or epinephrine (*p* = 0.001). Indeed, norepinephrine was used in 59% of patients with mottling vs. 48% in patients without (*p* = 0.008). Epinephrine was used twice more often in patients with mottling compared to patients without mottling (18.4% vs. 9.7%, *p* = 0.001).

Another major difference was the use of red blood cell transfusion, which was much more frequent in patients with mottling (21.6 vs. 15%, *p* = 0.03) despite equal levels of hemoglobin in both groups at admission. Regarding organ support, the need for invasive respiratory support was significantly higher in patients with mottling (50.2% vs. 30.1%, *p* < 0.001) and likewise for the need for RRT (19.9% vs. 12.4%, *p* = 0.09). However, the need for mechanical circulatory support (MCS) was similar in both groups.

Interventions such as radiofrequency ablation during hospitalization were less frequent in patients with mottling than patients without (0.8% vs. 3.4%, *p* = 0.047).

### Thirty-day and 1-year outcomes and correlates

The median length of stay in hospital was significantly higher in patients exhibiting mottling, with 19 vs. 16 days (*p* = 0.033). There was no difference between groups regarding discharge mode after hospitalization.

**Fig. 1:**
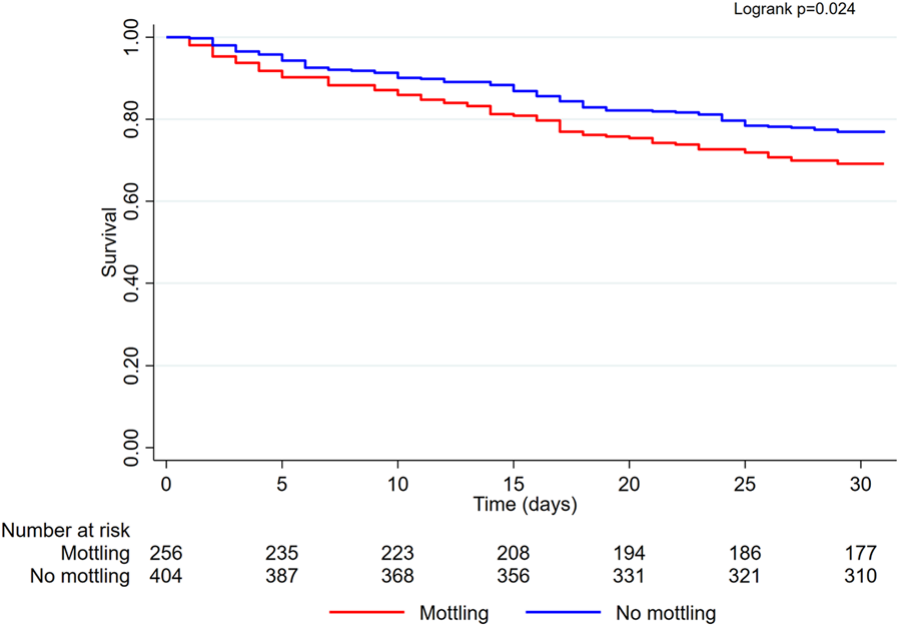
Kaplan–Meier curve showing early and long-term mortality in cardiogenic shock according to the presence of mottling at admission

The mortality rate at 30 days and 1 year were significantly increased in patients with mottling compared to patients without (31% vs. 23%, *p* = 0.031 and 54% vs. 42%, *p* = 0.003, respectively) (Fig. 1 and Additional file 1: Fig. S1).

**Fig. 2:**
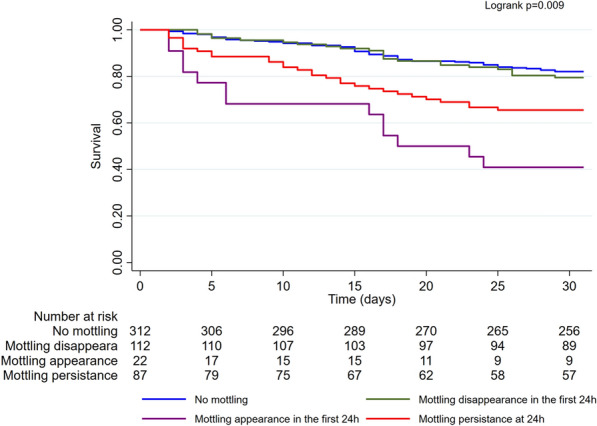
Kaplan–Meier curve showing early and long-term mortality in cardiogenic shock in the subgroup of patients who were still alive after 24 h, according to the presence of mottling at admission and its evolution at 24 h. Survival in the subgroup of patients who were still alive after 24 h according to the presence of mottling at admission and its evolution at 24 h. The “No mottling” group corresponds to patients without mottling at admission or at 24 h. The “mottling disappearance” corresponds to patients with mottling at admission that disappears at 24 h. The “mottling appearance” group corresponds to patients without mottling at admission in which mottling appears at 24 h. The “mottling persistence” group corresponds to patients with mottling at admission and mottling persistence at 24 h

Multivariate analyses identified as factors associated with 30-day mortality in patients with mottling: low LVEF (HR: 1.03; *p* = 0.05), low glomerular filtration rate (HR: 1.14; *p* = 0.036), low hemoglobin (HR: 1.15; *p* = 0.009), older age (for 1 year) (HR: 1.3; *p* = 0.03), current smoking (HR: 2.8; *p* < 0.01), history of ischemic stroke (HR: 2.92; *p* = 0.01), a mechanical trigger of CS (HR: 3.35; *p* = 0.022) (Fig. 2). A history of cardiac disease was associated with better outcomes (HR: 0.41; *p* = 0.001).

**Fig. 3:**
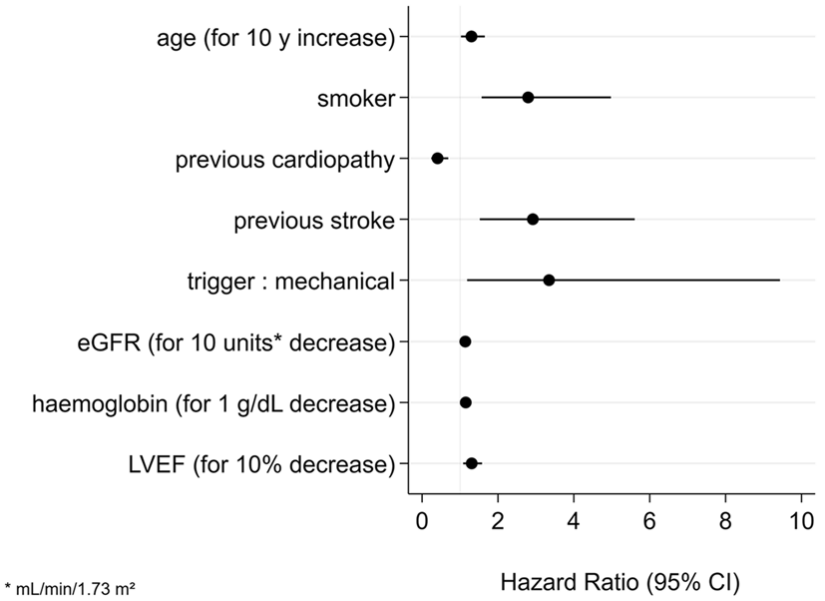
Forest plot of factors at admission associated with 30-day mortality in patients with mottling—multivariate analysis 30-day mortality

In the subgroup of patients who were still alive after 24 h (Fig. 3), the prognosis of patients whose mottling had regressed was comparable to that of patients who never had mottling (HR = 1.15 [0.71–1.86], *p* = 0.58 compared with patients who never had mottling). Conversely, the appearance or the persistence of mottling at 24 h was a poor prognostic factor (respectively, HR = 4.68 [2.56–8.57]; *p* < 0.001, and HR = 2.22 [1.42–3.46]; *p* < 0.001).

Additional file 2: Fig. S2 illustrates the respective weight of mottling and arterial lactate level at admission on mortality: patients without mottling and with arterial lactate < 4 mmol/L had a significantly higher probability of survival as compared with other groups of patients (HR = 0.59 [0.43–0.81], *p* = 0.001). No difference was found regarding the probabilities of 30-day survival between the three other groups of patients, i.e., patients without mottling and arterial lactate ≥ 4 mmol/L, patients with mottling and with arterial lactate < 4 mmol/L, and patients with mottling and with arterial lactate ≥ 4 mmol/L.

Additional files 3 and 4: Figs. S3 and S4 illustrate the composite endpoint of 30-day mortality or the need for acute mechanical circulatory support as endpoints. Additional file 5: Fig. S5 shows the integration of lactate level and mottling, in a sub-population, in which these parameters can be assessed at admission and 24 h.

## Discussion

To date, this analysis of the FRENSHOCK registry is the first analysis of mottling in CS based on a post hoc analysis of a large prospective register of unselected CS. Findings from this validation cohort confirm preliminary results from a previous smaller cohort of patients in cardiogenic shock [9].

Foreseeing the survival of patients with CS at the time of admission is challenging, requiring collection, integration, and analysis of complex clinical manifestations and biomarkers while managing the precarious hemodynamic status. In this pragmatic prospective observational study of patients with CS, we examined the effects of skin mottling assessment during a simple physical examination on survival and other relevant outcomes. The main results are that mottling was present at admission in almost 39% of patients in CS, patients admitted with mottling presented a significantly higher length of stay (19 vs. 16 days, *p* = 0.033), a higher 30-day mortality rate (31% vs. 23.3%, *p* = 0.031), and significantly higher mortality at 1-year (54% vs. 42%, *p* = 0.003). These results were consistent with previous studies based on Forrester's perfusion/congestion profiles, clinically adapted by Nohria et al*.* [24], showing that CS's main clinical presentations are mostly wet-cold (∼65%) and dry-cold (∼30%) (“cold” meaning hypoperfusion) [8, 25]. Of note, however, mottling as a clinical sign of end-organ hypoperfusion was not part of the parameters initially described by Nohria et al*.* [24].

The skin, as a conveniently accessible organ, offers the opportunity for easy assessment of local microcirculatory perfusion through observable changes in skin color, i.e., mottling [19]. The pathophysiological underpinnings of hypoperfusion in skin mottling remain incompletely understood [21, 26]. However, studies suggest that the foremost causative mechanism behind diminished blood perfusion is local vasoconstriction caused by sympathetic neuro-activation [27]. Further contributors to impaired microcirculatory flow may include local endothelial dysfunction [28], leukocyte adhesion, platelet activation, and fibrin deposition [19].

Although mottling has been known by physicians for decades [29], it was only in 2011 that Ait-Oufella et al*.*, developed an original clinical score of skin mottling, based on the extension of mottling around the knee (ranging from 0 to 5), allowing a more reliable assessment [30]. The reproducibility of this score was excellent with very good agreement between observers [30]. Based on this scoring system, studies found that mottling score measured 6 h after initial resuscitation in ICU, is a strong predictor of mortality in patients with septic shock [30, 31] but also among all critically ill patients admitted in ICU [32]. A decrease in mottling score during the first 6 h of resuscitation has also been significantly associated with better outcomes in septic shock [33]. Therefore, in 2014, an expert task force of the European Society of Intensive Care Medicine (ESICM) recommended assessing abnormal skin perfusion in their consensus on circulatory shock and hemodynamic monitoring [34].

The primary aim in the management of CS is to restore macrocirculation parameters such as SAP, mean arterial pressure (MAP), and CI. However, some studies have highlighted that up to 45% of patients who die of CS have a restored CI, explaining why optimization of macrocirculatory parameters alone may not be sufficient [35]. This observation may be partly due to organ perfusion disorders that extend beyond the macrocirculation and subsequently lead to multiple organ failures [36]. The state in which the main macrocirculation parameters such as SAP, MAP, and CI are restored, while microcirculation parameters remain impaired, is referred to as "loss of hemodynamic coherence" [37].

Here, past medical history was similar between groups independently of skin mottling (except for a higher rate of already implanted pacemakers or defibrillators in patients without skin mottling). However, in the multivariate analysis, a history of cardiac disease was associated with lower mortality at day 30 in patients with mottling at admission. One hypothesis that might explain this unexpected result, would be because of a vascular adaptation in patients with chronic heart failure [38]. Moreover, a lower prescription of aldosterone antagonists in medical history was also found in patients with mottling. Although there are limited data to date, these results might be explained by the deleterious effect of aldosterone on macro and microcirculation, which have been shown both in pre-clinical animal models [39, 40] and in humans [41, 42].

Clinical presentation and baseline echocardiography were significantly different whether the patient with CS had mottling or not, with higher non-compliance as a trigger of CS, higher heart rate, lower SAP and diastolic arterial pressure, and more severe aortic stenosis among patients with mottling at admission.

In-hospital management significantly differs since catecholamines, inotropes, volume expansion, transfusion, and organ support (invasive ventilation and RRT) were more often used in patients with mottling than in patients without. This was probably due to greater severity, with more mixed circulatory shocks combining a vasoplegic component with the pre-existing cardiogenic one, and also with greater end-organ hypoperfusion requiring more organ support.

Some may attribute these findings to the higher doses of vasopressors in the most severe patients’ group, however, a recent study has shown that mottling remains an independent high prognostic marker regardless of the dose of vasopressors in septic shock [33].

In the subgroup of patients who were still alive after 24 h, the prognosis of patients whose mottling had regressed at 24 h was comparable to that of patients who never had mottling. Whereas, in this subgroup, the appearance or persistence of mottling at 24 h was a poor prognostic factor. However, this subgroup analysis carries the inherent risk of immortal time bias [43].

Simple signs of peripheral tissue perfusion, such as mottling or CRT, could be of interest to guide hemodynamic management in CS. A recent large, randomized control clinical trial, the ANDROMEDA-SHOCK trial, suggested that CRT can be used to guide early resuscitation in septic shock, performing as well as lactate levels [44]. A Bayesian reanalysis of this study even showed that peripheral perfusion-targeted resuscitation may result in lower mortality and faster resolution of organ dysfunction when compared with a lactate-targeted resuscitation strategy [45].

Thus, the main finding of this study is that a simple clinical parameter such as mottling may markedly predict 30-day mortality in CS. As suggested by Additional file 2: Fig. S2, patients presenting mottling at admission have almost the same mortality as patients with an arterial lactate level ≥ 4 mmol/L. Thus, mottling may also be integrated with other variables to develop practical tools for risk assessment of short-term mortality for patients with CS, such as the Cardiogenic Shock Score [46] or the CardShock score [47], to help clinicians in their decision-making processes for MCS indications [48]. Thus, further prospective research is warranted to confirm how using mottling could be integrated in cardiogenic shock management.

As in any observational study, our analysis has limitations. Data from patients who died before informed consent was obtained were not collected and recorded in the database because of administrative regulations. Thus, it cannot be excluded that the most severe patients i.e., with several comorbidities, frailty, or multiple end-stage organ failure could not have been admitted to ICU/ICCU for futility or have been deceased before inclusion. This could be a source of bias resulting in an underestimation of mortality. *A confounding bias cannot be eliminated; indeed we cannot exclude that therapeutic management was not guided by peripheral tissue hypoperfusion.* The mottling data collected in FRENSHOCK consisted only of the presence or absence of mottling assessed by a senior physician once per day, without information on the intensity and extent of mottling, i.e., the mottling score. It would have been interesting in our study to evaluate this score more closely to better assess the impact of the intensity and modulations of this mottling score on the outcomes. Dark skin patients were not included only because accurate clinical evaluation of mottling is difficult to assess in this population. Another limitation to mention is that SCAI SHOCK Stage Classification was not used for the CS severity classification given that this score was not yet available at the time of the study, since it was published recently in 2019 [49] and updated in 2022 [50]. Finally, mottling cannot be generalized to all patients because dark skin severely limits the ability to properly assess this clinical sign [30].

## Conclusion

In this prospective multicenter observational study of critically ill patients with cardiogenic shock, our data confirm that skin mottling at admission in patients with cardiogenic shock was statistically associated with prolonged length of stay, and higher 30-day and 1-year mortalities.

Mottling is a simple non-invasive, priceless tool allowing a real-time assessment of microcirculation at bedside, which seems to be strongly associated with the outcome. Our results suggest that the presence of skin mottling and its evolution should be closely monitored while managing patients with cardiogenic shock. Further prospective research is, however, warranted to define the most effective way to integrate it into the early management of cardiogenic shock.

### Supplementary Information

**Additional file 1: Fig. S1**. Kaplan–Meier curve showing long-term mortality in cardiogenic shock according to the presence of mottling at admission.

**Additional file 2: Fig. S2**. Survival according to mottling and arterial lactate level at admission.

**Additional file 3: Fig. S3**. Kaplan–Meier curve showing 30-day mortality or the need for acute mechanical circulatory support in cardiogenic shock according to the presence of mottling at admission.

**Additional file 4: Fig. S4**. Kaplan–Meier curve showing 30-day mortality or the need for acute mechanical circulatory support in cardiogenic shock in the subgroup of patients who were still alive after 24 h, according to the presence of mottling at admission and its evolution at 24 h.

**Additional file 5: Fig. S5**. Kaplan–Meier curve showing 30-day mortality, according to the lactate level and the presence of mottling at admission and their evolutions at 24 h (*n* = 270).

## Data Availability

All summarized data are available upon request.

## Abbreviations

CI: Cardiac index
CRT: Capillary refill time
CS: Cardiogenic shock
ESICM: European Society of Intensive Care Medicine
IABP: Intra-aortic balloon pump
ICCU: Intensive cardiac care unit
ICU: Intensive care unit
IDF: Incident Dark-Field
LVEF: Left ventricular ejection fraction
MAP: Mean arterial pressure
MCS: Mechanical circulatory support
PCI: Percutaneous coronary intervention
RRT: Renal replacement therapy
SAP: Systolic arterial pressure
SDF: Sidestream Dark-Field
TAPSE: Tricuspid annular plane systolic excursion
VA-ECMO: Venoarterial-extracorporeal membrane oxygenation
