# Supplementary figures and images for: Mottling as a prognosis marker in cardiogenic shock
Source: Ann Intensive Care. 2023 Sep 6;13:80. doi: 10.1186/s13613-023-01175-0 (PMC10482815; doi:10.1186/s13613-023-01175-0)

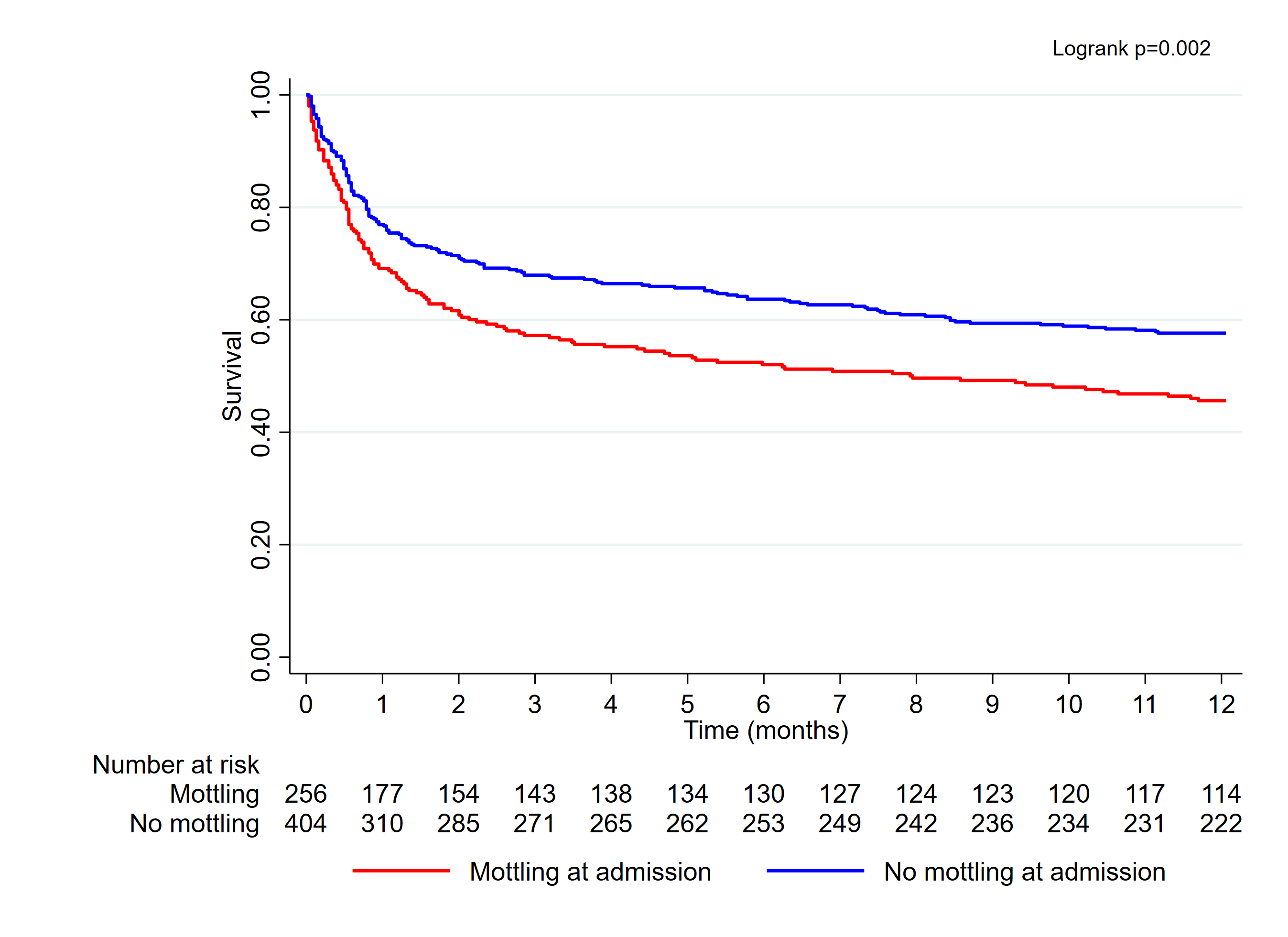

Supplement: Supplementary file 1 — Additional file 1: Fig. S1. Kaplan–Meier curve showing long-term mortality in cardiogenic shock according to the presence of mottling at admission. [file 13613_2023_1175_MOESM1_ESM.tif]

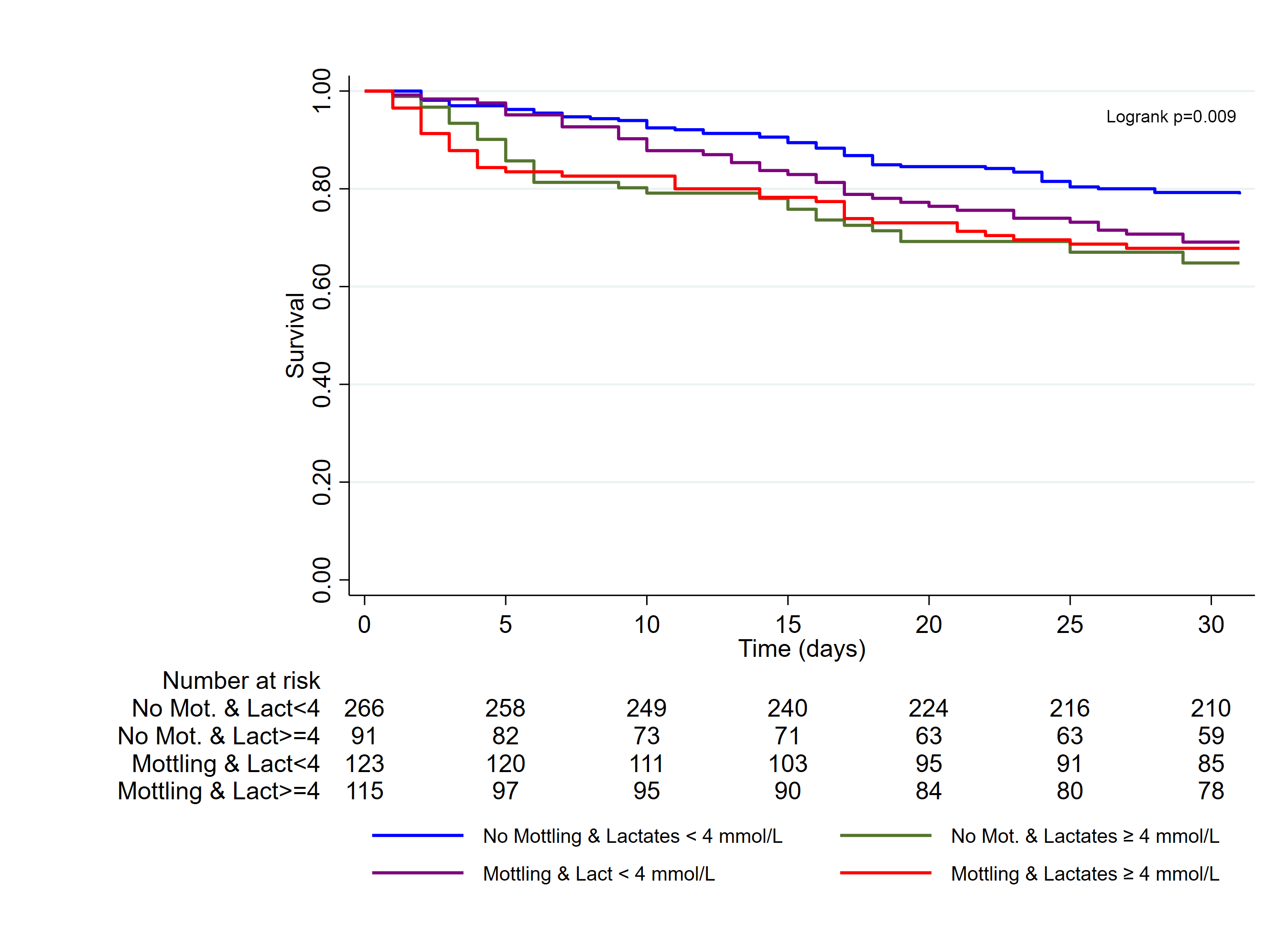

Supplement: Supplementary file 2 — Additional file 2: Fig. S2. Survival according to mottling and arterial lactate level at admission. [file 13613_2023_1175_MOESM2_ESM.tif]

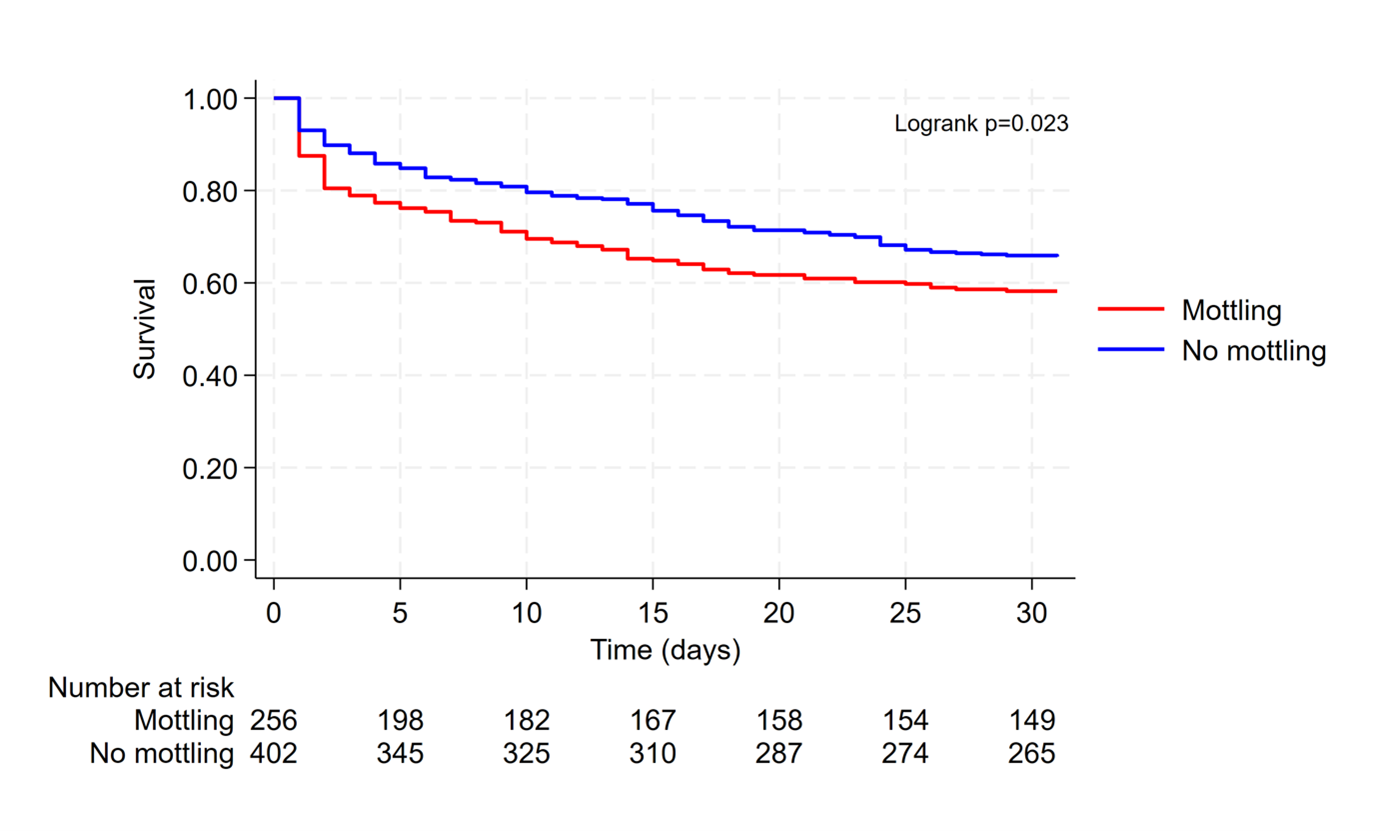

Supplement: Supplementary file 3 — Additional file 3: Fig. S3. Kaplan–Meier curve showing 30-day mortality or the need for acute mechanical circulatory support in cardiogenic shock according to the presence of mottling at admission. [file 13613_2023_1175_MOESM3_ESM.tif]

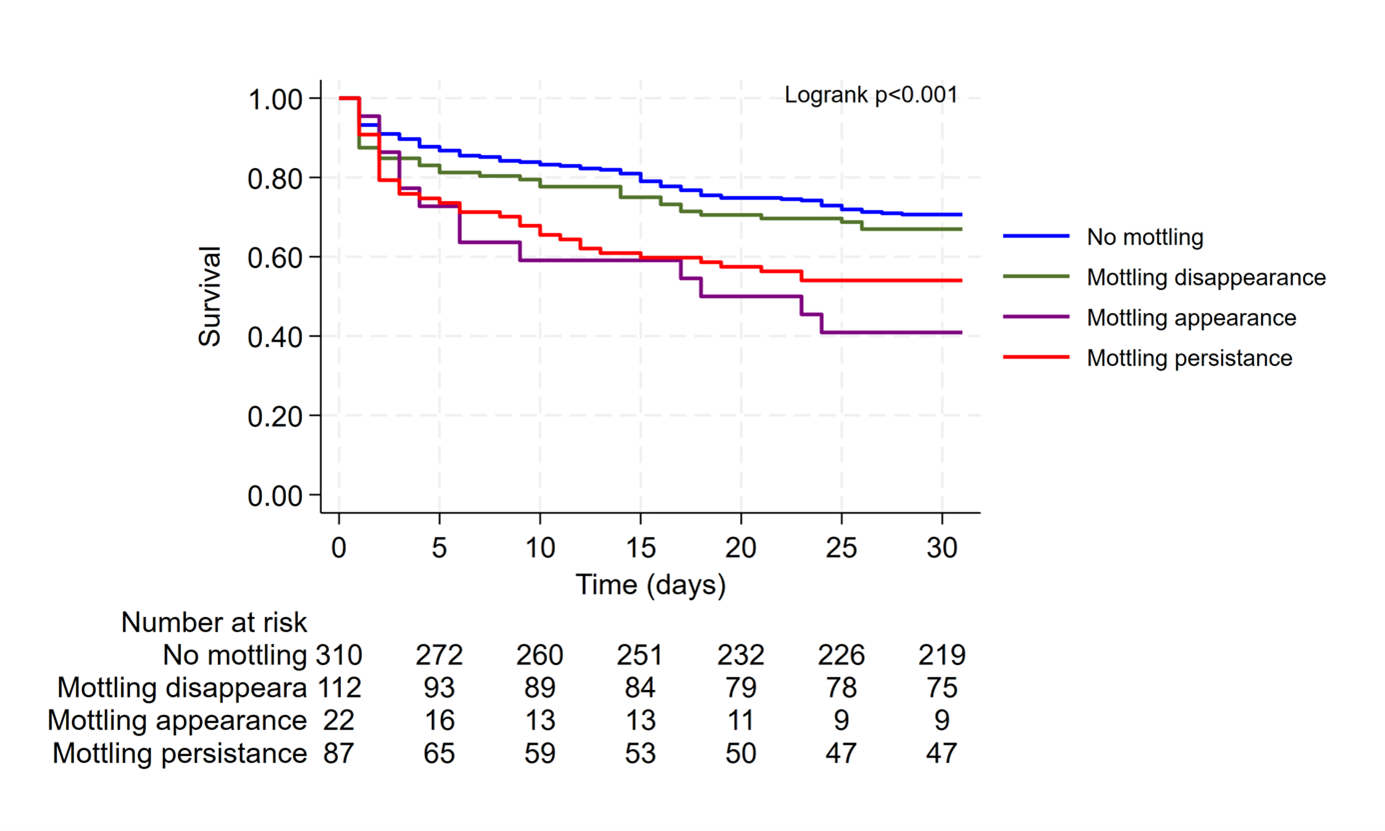

Supplement: Supplementary file 4 — Additional file 4: Fig. S4. Kaplan–Meier curve showing 30-day mortality or the need for acute mechanical circulatory support in cardiogenic shock in the subgroup of patients who were still alive after 24 h, according to the presence of mottling at admission and its evolution at 24 h. [file 13613_2023_1175_MOESM4_ESM.tif]

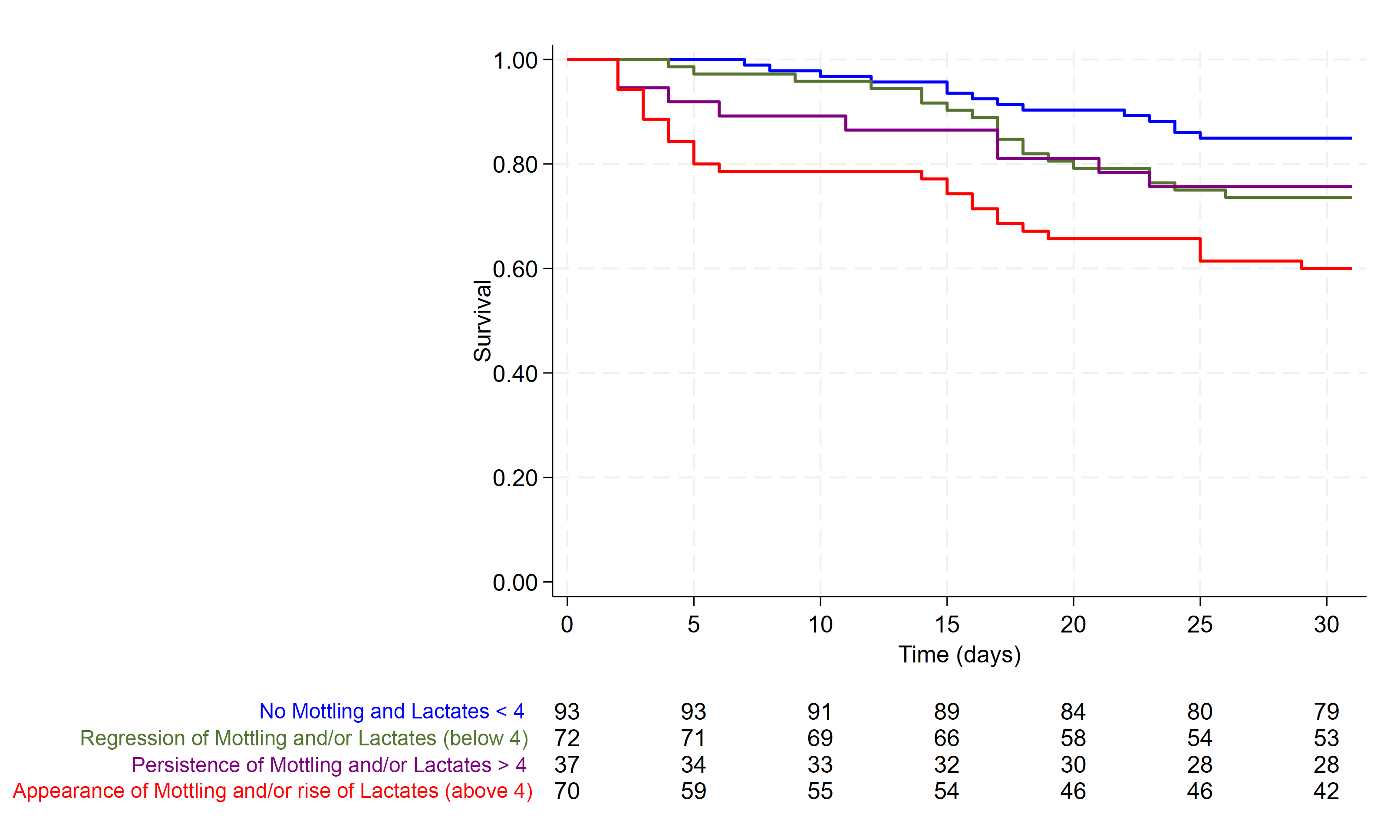

Supplement: Supplementary file 5 — Additional file 5: Fig. S5. Kaplan–Meier curve showing 30-day mortality, according to the lactate level and the presence of mottling at admission and their evolutions at 24 h (n = 270). [file 13613_2023_1175_MOESM5_ESM.tif]
